# Passive leg raising cannot predict volume responsiveness in septic shock patients having cardiac arrhythmia

**DOI:** 10.1186/cc14265

**Published:** 2015-03-16

**Authors:** P Ratanawatkul, A Wattanathum

**Affiliations:** 1 Srinagarind Hospital, Khon Kaen, Thailand; 2 Phramongkutklao Hospital, Bangkok, Thailand

## Introduction

The passive leg raising test (PLRT) is a self-volume challenge used in order to predict volume responsiveness (VR) in both spontaneous and mechanically ventilated critically ill patients. However, there were small numbers of arrhythmic patients included in previous studies. Therefore, the accuracy of the PLRT for prediction of VR in arrhythmic patient is still inconclusive. We hypothesized that the PLRT can predict VR in mechanically ventilated patients having cardiac arrhythmia.

## Methods

Mechanically ventilated patients having cardiac arrhythmia who have been considered for volume expansion were recruited in this prospective study. Each patient was sedated, paralyzed and monitored with a central venous catheter and a thermistor-tipped femoral arterial VolumeView catheter connected to the EV1000 monitor. We assessed hemodynamic changes after PLRT via a pulse wave contour analysis method. Then we compared it with hemodynamic changes after volume expansion (NSS 500 ml in 15 minutes) via the transpulmonary thermodilution (TPTD) method.

## Results

A total of 17 patients were included in this study. Six patients were volume responders (TPTD cardiac index change ≥15%). A PLRT change cardiac index ≥10% from the pulse wave contour analysis method had predicted VR with a sensitivity of 50%, a specificity of 72.7% and an area under the ROC curve of 0.591 (*P *= 0.546).

## Conclusion

The PLRT may not be used for prediction of VR in mechanically ventilated patients having cardiac arrhythmia; however, further and larger studies are needed to confirm this finding.
